# The Role of the Individual Bundles of the Deltoid Ligament in Multidirectional Stability and Articular Contact Pressure of the Ankle Joint: A Finite Element Analysis

**DOI:** 10.3390/bioengineering13020145

**Published:** 2026-01-27

**Authors:** Yuandong Li, Xiaoxi Ji, Qingqing Yang, Huizhi Wang, Cheng-Kung Cheng

**Affiliations:** 1 School of Biomedical Engineering and Engineering Research Center for Digital Medicine of the Ministry of Education, Shanghai Jiao Tong University, Shanghai 200030, China; liyuandong@sjtu.edu.cn (Y.L.); yangqq1@sjtu.edu.cn (Q.Y.); 2 Department of Sports Medicine, Huashan Hospital, Fudan University, Shanghai 200040, China; jixiaoxi87@163.com; 3 Center for Intelligent Medical Equipment and Devices, School of Biomedical Engineering, Division of Life Sciences and Medicine, University of Science and Technology of China, Hefei 230026, China; 4 Suzhou Institute for Advanced Research, University of Science and Technology of China, Suzhou 215123, China

**Keywords:** deltoid ligament, ankle instability, ankle joint biomechanics, finite element analysis

## Abstract

The deltoid ligament (DL) is the primary stabilizer of the medial ankle; however, a limited understanding of the functional roles of its various bundles hinders rational surgical decision-making. This study aims to investigate the roles of individual DL bundles in maintaining ankle stability and articular contact pressure and thus seeks to guide decisions on whether reconstruction is required for specific injuries. A validated finite element foot model was used to simulate isolated and multiple deficiencies in the DL bundle. The articular displacements, rotations, and peak talar cartilage contact pressure were evaluated under anterior drawer force and under internal–external rotation, eversion, and plantarflexion–dorsiflexion moments. Compared with the intact model, anterior tibiotalar ligament (ATTL) deficiency resulted in the greatest anterior drawer displacement (increase: 29%). Talonavicular ligament (TNL) deficiency caused the largest internal–external rotation and plantarflexion (increases in external rotation: 69%; in internal rotation: 10%; in plantarflexion: 32%). Tibiocalcaneal ligament (TCL) deficiency caused the largest eversion (increase: 93%). Deep posterior tibiotalar ligament (dPTTL) deficiency caused the largest dorsiflexion (increase: 68%). The maximum talar cartilage contact pressure occurred in the TNL-deficient model under the plantarflexion condition. In conclusion, individual DL bundles exhibit specific functions in terms of controlling multidirectional ankle stability—the ATTL, TNL, TCL, and dPTTL are the primary stabilizers for anterior translation, rotation/plantarflexion, eversion, and dorsiflexion, respectively. These findings provide a biomechanical rationale for personalized surgical strategies. When comprehensive DL reconstruction is not feasible, clinicians can prioritize the reconstruction of specific bundles according to the patient’s instability severity and functional demands across degrees of freedom.

## 1. Introduction

The deltoid ligament (DL) is the primary structure that maintains the medial stability of the ankle joint by preventing excessive internal–external rotation, eversion, plantarflexion, dorsiflexion, and anterior movement [1,2,3,4]. Anatomical investigation indicates that the DL consists of two layers, comprising four superficial and two deep bundles [5,6]. While conservative management is often the initial treatment for DL injury [7,8,9], its failure can lead to chronic medial instability [10] and a long-term risk of osteoarthritis [11]. This instability may result in further cumulative damage to the DL complex, ultimately leading to overall structural laxity [12] and requiring reconstruction surgery. However, there is currently no consensus on the optimal surgical technique [13]. Although ideal anatomical reconstruction would involve repairing all injured bundles, this is often impractical due to the inherent technical challenges [12]. As a result, many procedures are effectively selective, making it crucial to identify which DL bundles are most important for restoring ankle stability and contact mechanics. However, the relative biomechanical contributions of individual DL bundles remain insufficiently defined.

Early cadaveric studies primarily evaluated the effects of the DL on ankle stability and articular contact pressure by transecting it as a single entity [14,15] or transecting it in layers [2,3,16,17], yet evidence regarding the functional contribution of individual bundles remains scarce. This is largely attributable to the limited understanding of its intricate anatomical subdivisions that prevailed at the time [18,19,20], as well as the high level of complexity involved in conducting bundle-level assessments in cadaveric experiments [21]. In contrast, finite element analysis offers a more versatile approach, making it possible for researchers to selectively remove and restore individual ligaments within the model, and enabling accurate assessment of the specific biomechanical function of each DL bundle. However, these finite element analyses have focused only on two particular bundles (the TCL and ATTL) [22,23], which may be due to incomplete representation of the DL complex in the deployed models, limiting their ability to support systematic analyses of all six individual bundles.

Additionally, previous studies evaluated DL performance under only a limited set of conditions [14,15] (eversion, external rotation, and the anterior drawer test), with relatively little testing during plantarflexion–dorsiflexion and internal rotation. However, recent studies indicate that these conditions are also critical, as plantarflexion and dorsiflexion constitute the predominant ankle motions during the gait cycle [24] and the DL plays a previously underappreciated role in maintaining internal rotational stability [25,26].

Thus, this study aimed to use a validated finite element foot model to systematically analyze the roles played by individual DL bundles in maintaining ankle stability against anterior translation, internal rotation, external rotation, eversion, plantarflexion, and dorsiflexion, as well as in maintaining normal articular contact pressure. We hypothesized that the contributions of individual bundles to ankle stability and articular contact pressure vary across different degrees of freedom. By defining these bundle-specific roles, we expect to establish a “priority list” for surgical decision-making, guiding the selective reconstruction of key ligaments based on the patient’s unique instability pattern and functional demands, particularly when comprehensive DL reconstruction is not feasible.

## 2. Materials and Methods

### 2.1. Study Design

**Figure 1:**
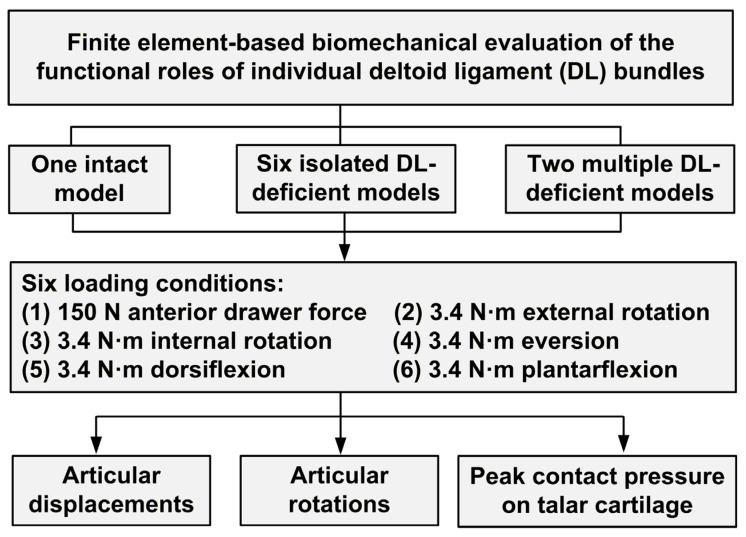
A flowchart of the study design.

This study utilized a validated 3D finite element model of the human foot to systematically analyze the biomechanical functions of individual deltoid ligament bundles (Figure 1). A total of nine models were established, comprising one intact model and eight DL-deficient models (including both isolated and multiple DL-deficient models). All models were subjected to the following six loading conditions: a 150 N anterior drawer force and 3.4 N·m moments simulating internal–external rotation, eversion, and plantarflexion–dorsiflexion. Subsequently, articular displacements, rotations, and peak talar cartilage contact pressures were quantified to identify the bundles responsible for maintaining biomechanical function across different degrees of freedom.

### 2.2. Finite Element Model of the Foot

The development and validation procedures for the finite element foot model used in this study are described in detail in our previous publications [26]. The institutional ethics committee approved all model development procedures (approval number: 2020-1123). A brief overview of the model is provided below.

**Figure 2:**
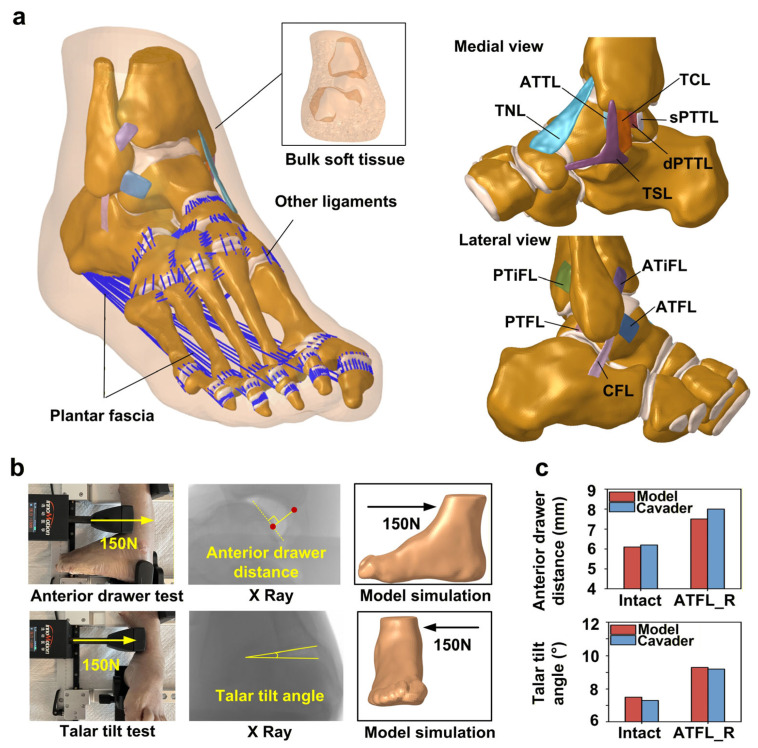
Establishment and validation of the finite element model of the human foot. (**a**) Overview of the finite element foot model. Talonavicular ligament (TNL); tibiospring ligament (TSL); anterior tibiotalar ligament (ATTL); tibiocalcaneal ligament (TCL); superficial posterior tibiotalar ligament (sPTTL); deep posterior tibiotalar ligament (dPTTL); anterior talofibular ligament (ATFL); posterior talofibular ligament (PTFL); calcaneofibular ligament (CFL); anterior tibiofibular ligament (ATiFL); posterior tibiofibular ligament (PTiFL); (**b**) illustration of biomechanical validation; (**c**) results of biomechanical validation.

The model includes bones, cartilage, ligaments, the plantar fascia, and the surrounding bulk soft tissue (Figure 2a). The three-dimensional geometries of eleven ankle ligaments were included in the model, comprising five lateral ligaments (the anterior talofibular ligament (ATFL), posterior talofibular ligament (PTFL), calcaneofibular ligament (CFL), anterior tibiofibular ligament (ATiFL), and posterior tibiofibular ligament (PTiFL)) and six medial ligaments (the talonavicular ligament (TNL), tibiospring ligament (TSL), anterior tibiotalar ligament (ATTL), tibiocalcaneal ligament (TCL), superficial posterior tibiotalar ligament (sPTTL), and deep posterior tibiotalar ligament (dPTTL)). The three-dimensional structures were meshed into first-order, four-node tetrahedral elements. Cartilage interactions were modeled as frictionless sliding contacts. Ligaments were fixed to their bony insertions to maintain consistent displacement, and binding constraints were implemented between the cartilage and adjacent bone surfaces, as well as between bone and the encasing soft tissue. Details of material properties are provided in Table 1.

For biomechanical validation, a Ligs digital joint measurement device (Innomotion Inc., Shanghai, China) and an X-ray imaging system were used to perform anterior drawer tests (ADTs) and talar tilt tests on the same cadaveric specimen used for model development. During ADT, the calf and heel were fixed. At the same time, a 150 N anterior force was applied to the tibia at a loading rate of 3 N/s, with simultaneous radiographic imaging (Figure 2b). The device was then adjusted to apply a medial force to the tibia using the same loading parameters, again with concurrent imaging. Subsequently, the ATFL was surgically transected, and all procedures were repeated. Two parameters were quantified using RadiAnt DICOM Viewer software (version 5.0.2, Medixant, Poznan, Poland): (1) the anterior drawer distance, defined as the vertical distance between the posterior margins of the tibia and talus, and (2) the talar tilt angle, defined as the angle between the tibial and talar articular surfaces. Each measurement was repeated three times, and the mean value was used for analysis to reduce variability. The finite element model was subsequently subjected to the same test conditions for comparison. The anterior drawer distances and talar tilt angles predicted by the finite element model before and after ATFL transection closely matched the experimental measurements, exhibiting discrepancies within 6.6% (Figure 2c).

The skin, adipose tissue, fascia, and musculotendinous structures overlying the ankle joint were carefully dissected to fully expose the ligamentous complexes for anatomical validation. A vernier caliper with an accuracy of 0.1 mm was used to measure the length, width, and thickness of each ligament. Given that the DL contains both superficial and deep layers, the bundles in the superficial layer were measured first, followed by careful separation to examine the deeper structures. The same methodology was used to obtain dimensional data for each ligament in the finite element model and to compare it with the experimental results. Anatomical validation showed that ligament dimensions obtained from the model closely matched the anatomical measurements, with maximum differences of 1.8 mm in length, 0.6 mm in width, and 0.2 mm in thickness.

### 2.3. Establishment of DL-Deficient Models

DL-deficient models were established by removing the corresponding ligaments from the intact model. The established DL-deficient models were as follows: (1) isolated DL-deficient models: rupture of the TNL (TNL_R); rupture of the TSL (TSL_R); rupture of the TCL (TCL_R); rupture of the ATTL (ATTL_R); rupture of the sPTTL (sPTTL_R); rupture of the dPTTL (dPTTL_R); (2) multiple DL-deficient models: rupture of all superficial bundles (All superficial_R); rupture of all deep bundles (All deep_R). In addition to the intact model, nine models were included in this study.

### 2.4. Coordinate System Definition and Loading Conditions

**Figure 3:**
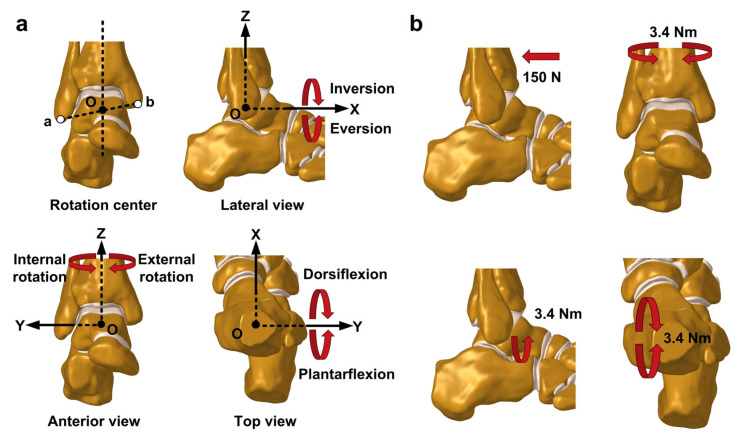
Coordinate system and loading conditions. (**a**) Establishment of the center of rotation and the coordinate system for the ankle joint, along with a schematic representation of everyday ankle movements; (**b**) simulations of anterior drawer, internal–external rotation, eversion, and plantarflexion–dorsiflexion conditions.

The coordinate system in the finite element model was established according to the method described by Brockett and Chapman [28] (Figure 3a). The center of rotation was defined as the midpoint between the fibular apex and the medial malleolar apex. The Y-axis passed through this center and was oriented perpendicular to the sagittal plane. The Z-axis also passed through the center and was aligned with the tibia’s longitudinal axis, perpendicular to the Y-axis. The X-axis was perpendicular to the other two axes.

The following loading conditions were simulated in this study (Figure 3b), all with the calcaneus fixed: (1) the anterior drawer test was simulated by applying a 150 N posteriorly directed force to the anterior edge of the tibia; (2) internal–external rotation conditions were simulated by applying a ±3.4 Nm moment to the tibia around the Z-axis; (3) the eversion condition was simulated by applying a 3.4 Nm moment to the tibia around the X-axis; and (4) plantarflexion–dorsiflexion conditions were simulated by applying a ±3.4 Nm moment to the tibia around the Y-axis. For each DL-deficient model, the articular rotations and displacements were recorded, along with the maximum contact pressure and its distribution in the talar cartilage.

## 3. Results

### 3.1. Effects of DL-Deficient Conditions on Anterior Stability and Talar Cartilage Contact Pressure

**Figure 4:**
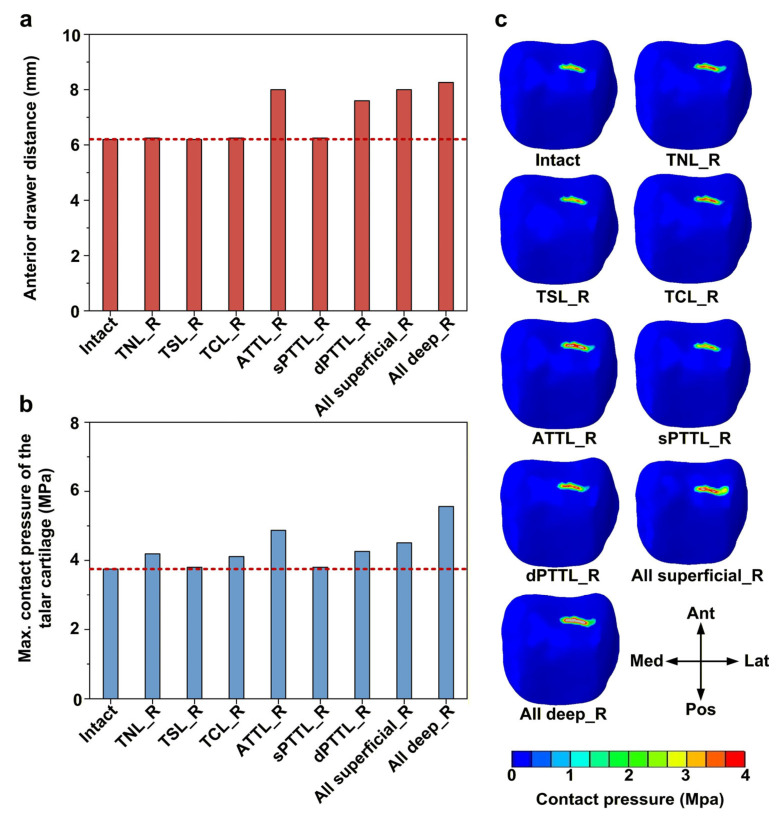
Biomechanical response of the ankle joint under anterior drawer force. (**a**) The anterior drawer displacement; (**b**) the maximum contact pressure of the talar cartilage; and (**c**) the contact pressure distribution of the talar cartilage. The red dashed line represents the value of the intact model.

In all isolated DL-deficient models, deficiencies of the anterior tibiotalar ligament (ATTL) and the deep posterior tibiotalar ligament (dPTTL) resulted in a pronounced increase in anterior drawer displacement compared with the intact model, with increases of 29% (8.01 vs. 6.20) and 23% (7.60 vs. 6.20), respectively (Figure 4a). Building on this, a complete deficiency of the two deep bundles caused a slight further increase in anterior drawer displacement, greater than that caused by isolated ATTL or dPTTL deficiencies, respectively. Accordingly, among all isolated DL-deficient models, ATTL deficiency caused the highest talar cartilage contact pressure, with an increase of 30% (4.87 vs. 3.75) compared with the intact model (Figure 4b). When both deep bundles were deficient, the talar cartilage contact pressure reached the highest value observed in this study, representing a 48% (5.56 vs. 3.75) increase relative to the intact model. Under anterior drawer force, the talar cartilage contact pressure in all models was predominantly concentrated on the anterolateral region of the talar dome (Figure 4c).

### 3.2. Effects of DL-Deficient Conditions on External Rotation Stability and Talar Cartilage Contact Pressure

**Figure 5:**
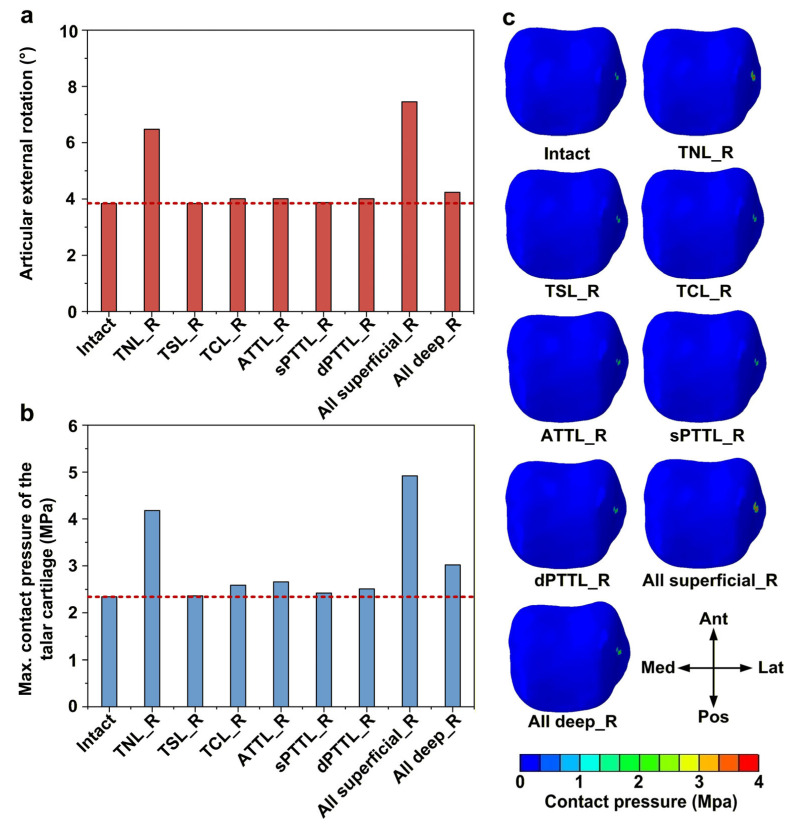
Biomechanical response of the ankle joint under external rotation moment. (**a**) The articular external rotations; (**b**) the maximum contact pressure of the talar cartilage; and (**c**) the contact pressure distribution of the talar cartilage. The red dashed line represents the value of the intact model.

Among the isolated DL-deficient models, the talonavicular ligament (TNL) deficiency produced the greatest external rotation in the ankle, with a 69% (6.47 vs. 3.83) increase relative to the intact model (Figure 5a). When all superficial DL bundles were deficient, the external rotation of the ankle exhibited a slight further increase and was 15% (7.44 vs. 6.47) higher than that in the isolated TNL deficiency. Consistent with these kinematic changes, isolated TNL deficiency also led to a substantial increase in the peak talar cartilage contact pressure, which increased by 79% (4.18 vs. 2.34) relative to the intact model (Figure 5b). When all superficial bundles were deficient, the peak talar cartilage contact pressure increased further, by 18% (4.92 vs. 4.18) compared with the isolated TNL-deficient model. All models showed a similar pattern of contact pressure distribution under external rotation moment (Figure 5c)—contact pressure was concentrated mainly in the mid-lateral region of the talar cartilage.

### 3.3. Effects of DL-Deficient Conditions on Internal Rotation Stability and Talar Cartilage Contact Pressure

**Figure 6:**
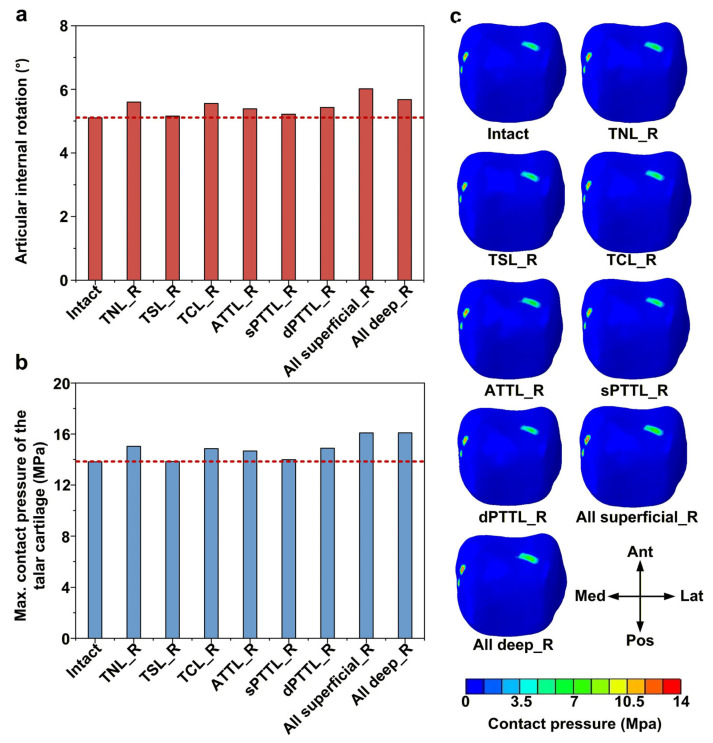
Biomechanical response of the ankle joint under internal rotation moment. (**a**) The articular internal rotations; (**b**) the maximum contact pressure of the talar cartilage; and (**c**) the contact pressure distribution of the talar cartilage. The red dashed line represents the value of the intact model.

All isolated DL-deficient models showed a 1–10% ((5.15–5.60) vs. 5.09) increase in internal rotation of the ankle compared with the intact model, with the talonavicular ligament (TNL) deficiency demonstrating the greatest increase (Figure 6a). The largest increase occurred when all superficial DL bundles were deficient, resulting in an articular rotation approximately 18% greater than that of the intact model. Similarly, in the isolated DL-deficient models, peak contact pressure in the talar cartilage increased by approximately 0.1–8.8% ((13.84–15.04) vs. 13.82) relative to the intact model (Figure 6b). When either all superficial or all deep DL bundles were deficient, the peak talar cartilage contact pressure reached the highest level in this series, roughly 16% (16.10 vs. 13.82) greater than that in the intact model. The pattern of contact pressure distribution in the talar cartilage was generally similar across all models (Figure 6c). The main contact pressure concentrations were located in the anteromedial region, the posterolateral region, and the upper right and lower left quadrants of the talar dome.

### 3.4. Effects of DL-Deficient Conditions on Eversion Stability and Talar Cartilage Contact Pressure

**Figure 7:**
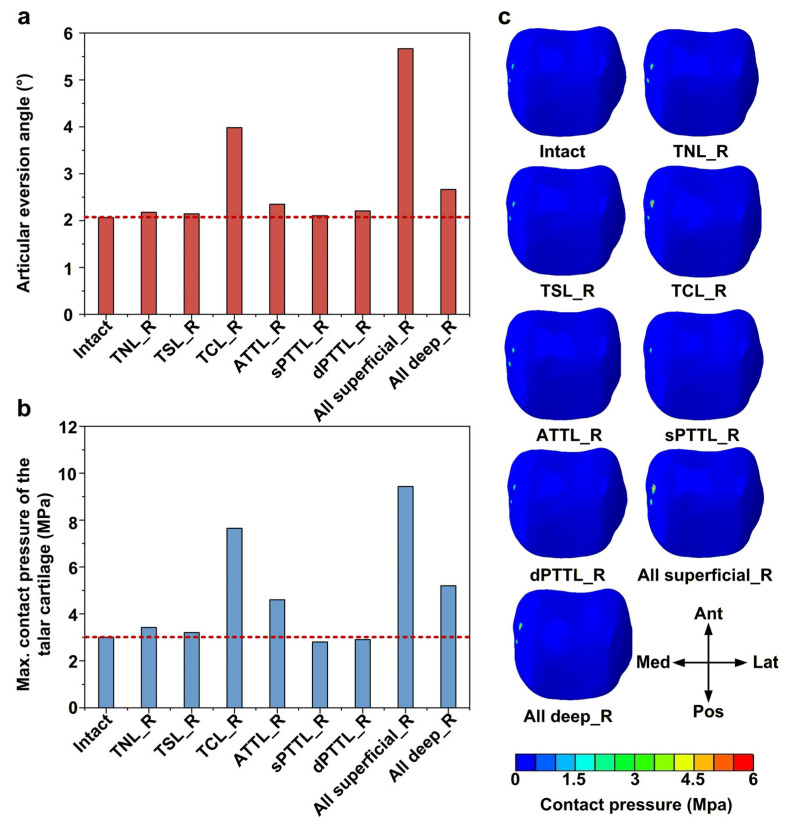
Biomechanical response of the ankle joint under an eversion moment. (**a**) The articular eversion angles; (**b**) the maximum contact pressure of the talar cartilage; and (**c**) the contact pressure distribution of the talar cartilage. The red dashed line represents the value of the intact model.

Among isolated DL-deficient models, the tibiocalcaneal ligament (TCL) deficiency resulted in the largest articular eversion angle, with a 93% (3.98 vs. 2.06) increase relative to the intact model (Figure 7a). Furthermore, when all superficial DL bundles were deficient, the eversion increased further to 2.7 times (5.66 vs. 2.06) that of the intact model and 1.4 times (5.66 vs. 3.98) that of the isolated TCL-deficient model. These changes in motion were accompanied by substantial increases in the peak contact pressure of the talar cartilage (Figure 7b). Isolated TCL deficiency increased the peak talar cartilage contact pressure to about 2.5 times (7.65 vs. 3.01) the value in the intact model. When all superficial DL bundles were deficient, this peak contact pressure increased further to about 3.1 times (9.43 vs. 3.01) the value in the intact model and 1.2 times (9.43 vs. 7.65) the value in the isolated TCL-deficient model. Under an eversion moment, the contact pressure in the talar cartilage was mainly concentrated on the medial side (Figure 7c).

### 3.5. Effects of DL-Deficient Conditions on Plantarflexion Stability and Talar Cartilage Contact Pressure

**Figure 8:**
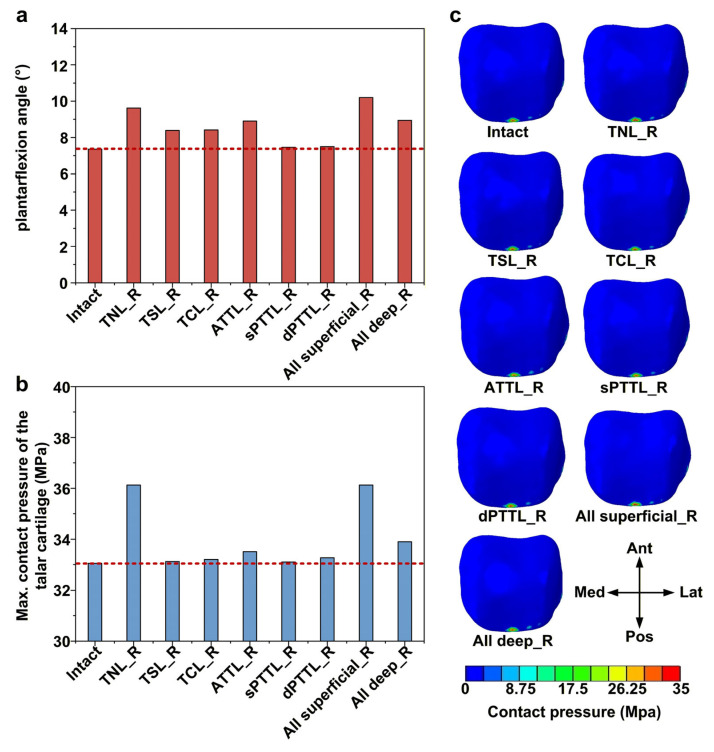
Biomechanical response of the ankle joint under a plantarflexion moment. (**a**) The articular plantarflexion angles; (**b**) the maximum contact pressure of the talar cartilage; and (**c**) the contact pressure distribution of the talar cartilage. The red dashed line represents the value of the intact model.

Among all isolated DL-deficient models, the talonavicular ligament (TNL) deficiency resulted in the largest articular plantarflexion, with an increase of about 31% (9.62 vs. 7.36) compared with the intact model (Figure 8a). The anterior tibiotalar ligament (ATTL) deficiency caused the second-largest increase in plantarflexion angle, with the angle elevated by approximately 21% (8.90 vs. 7.36) relative to the intact model. When all superficial DL bundles were deficient, the plantarflexion angle reached its highest value in this study, increasing by approximately 38% (10.19 vs. 7.36) relative to the intact model. Similarly, the TNL deficiencies led to marked increases in the peak contact pressure of the talar cartilage, by approximately 9% (36.13 vs. 33.05) compared with the intact model (Figure 8b). In all models, contact pressure in the talar cartilage was primarily concentrated at the posterior margin of the talar dome (Figure 8c).

### 3.6. Effects of DL-Deficient Conditions on Dorsiflexion Stability and Talar Cartilage Contact Pressure

**Figure 9:**
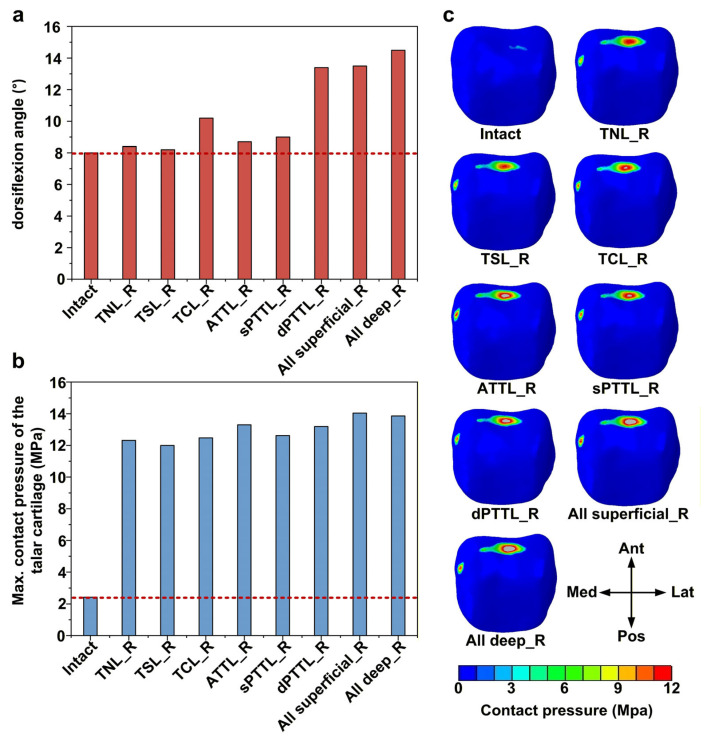
Biomechanical response of the ankle joint under a dorsiflexion moment. (**a**) The articular dorsiflexion angles; (**b**) the maximum contact pressure of the talar cartilage; and (**c**) the contact pressure distribution of the talar cartilage. The red dashed line represents the value of the intact model.

Among all isolated DL-deficient models, the deep posterior tibiotalar ligament (dPTTL) deficiency resulted in the largest articular dorsiflexion, with an increase of approximately 67% (13.4 vs. 8.01) compared with the intact model (Figure 9a). In addition, the tibiocalcaneal ligament (TCL) deficiency resulted in relatively large dorsiflexion, with approximately 27% (10.19 vs. 8.01) greater dorsiflexion than in the intact model. In all DL-deficient models, there were marked increases in peak talar cartilage contact pressure, to approximately 4.9–5.8 ((12.01–14.04) vs. 2.43) times that of the intact model (Figure 9b). In the intact model, the contact pressure was mainly concentrated in a narrow area at the anterior aspect of the talar dome (Figure 9c). By contrast, all DL-deficient models showed a different pattern of the contact pressure change, with clear expansion of the high-pressure region in the anterior talar dome and the appearance of a new high-pressure area in the anteromedial cartilage.

## 4. Discussion

This study demonstrates that the various bundles of the DL contribute differently to ankle stability across different degrees of freedom. The anterior tibiotalar ligament (ATTL) and the deep posterior tibiotalar ligament (dPTTL) were identified as the primary contributors to anterior stability. For rotational stability, the talonavicular ligament (TNL) plays a crucial role during internal–external rotation. The tibiocalcaneal ligament (TCL) is the most important during eversion. TNL and ATTL are the most important during plantarflexion, whereas dPTTL is crucial during dorsiflexion.

Under an anterior drawer force, the ATTL and dPTTL bundles contribute most to anterior stability. In anatomical terms, these two bundles belong to the deep component of the DL and are thicker yet shorter than the superficial bundles. In particular, the dPTTL is the thickest and shortest among all of the DL bundles [29]. This short, robust morphology may enable this bundle to exhibit less elastic deformation than other bundles under the same load conditions. In comparison, the ATTL deficiency resulted in greater anterior drawer displacement than the dPTTL deficiency, which may be related to its higher elastic modulus, which is approximately twice that of the dPTTL [30]. Previous studies have generally suggested that the translational motion of the talus within the mortise is primarily constrained by these two deep bundles [7,8], a finding consistent with our results.

Compared with the other DL bundles, the more horizontal fiber orientation of the TNL may enable it to withstand larger rotational moment components. A previous study reported that both the ATFL and DL were responsible for restricting internal–external ankle rotation [25]. Our study further demonstrated that, among the DL bundles, the TNL is the most effective in limiting internal–external rotation of the ankle. However, this finding differs from that of Palazzi et al., who reported that external rotation stability of the ankle is primarily maintained by the ATTL [22]. This discrepancy may be attributed to the exclusion of the TNL in their model, which likely resulted in a greater proportion of the external rotational torque being distributed to the ATTL.

The TCL provided the most significant contribution to eversion stability among all DL bundles, which is likely attributable to its fiber orientation, anatomical position, and mechanical properties. The TCL fibers are oriented almost parallel to the coronal plane. The eversion moment also acts within the coronal plane, enabling the TCL to bear a larger component of the eversion moment than the other bundles. Additionally, the more peripheral position of the TCL relative to the center of rotation [18] confers it with a longer moment arm, enabling it to generate a greater opposing moment to resist excessive eversion. Furthermore, the TCL possesses the highest Young’s modulus [27] and considerable thickness [21] among all DL bundles, which also enhances its ability to provide stable support. Previous studies have indicated that the superficial DL bundles provided the primary resistance to articular eversion [31,32]. In contrast, our findings further suggest that the TCL within the superficial layer makes the predominant contribution to eversion stability.

The ligaments located at the front of the ankle (TNL and ATTL) contribute more to stability during plantarflexion. In contrast, those at the back (dPTTL) and in the middle (TCL) contribute more to stability during dorsiflexion. This pattern is consistent with previous results showing that the anterior bundles elongated during plantarflexion, whereas the posterior bundles elongated during dorsiflexion [17,20,33]. In addition, early cadaveric studies reported that the length of the TCL remained nearly unchanged throughout ankle dorsiflexion–plantarflexion [34], suggesting that it may play only a limited role in constraining the motion of the ankle joint in the sagittal plane. In contrast, our results showed that the TCL-deficient model led to a large angular change during dorsiflexion, suggesting that the TCL also plays a significant role in maintaining ankle stability during dorsiflexion. This is also likely due to its high elastic modulus [27] and considerable thickness [21], which result in no significant change in strain even under loading. However, a recent cadaveric study [33] and a multi-body dynamic simulation [22] demonstrated that both the TCL and dPTTL experienced a substantial increase in tension during ankle dorsiflexion. These findings collectively provide strong support for our conclusions.

Additionally, our results indicate that the tibiospring ligament (TSL) and the superficial posterior tibiotalar ligament (sPTTL) provide limited contributions to ankle stability across the specified loading conditions. One existing study indicates that the TSL primarily works synergistically with the posterior tibial tendon to maintain the stability of the medial longitudinal arch, thereby preventing the development of adult-acquired flatfoot deformity [35,36]. In contrast, our study focuses on evaluating the rotational and translational stability of the ankle joint across various degrees of freedom. Consequently, the TSL did not demonstrate a significant direct mechanical contribution to the stability parameters selected in this study. Furthermore, previous studies have indicated that the sPTTL possesses the lowest Young’s modulus [27] among all DL bundles and is notably thin [26,29]. Such constraints in both anatomical morphology and material mechanical properties likely account for its minimal contribution to maintaining ankle joint stability.

We found that, as the degree of joint instability increased, the peak contact pressure on the talar cartilage increased correspondingly. This is attributable to greater articular rotations or translations, resulting in increased compression between the talus and adjacent bony structures. Some researchers have attributed cartilage damage to increased pressure on the joint surface [37]. MRI scans [38] of patients with chronic ankle instability have shown progressive degeneration in the medial and lateral regions of talar cartilage, as well as the anterior region of the talar dome. Our study also observed a similar trend: contact pressure was concentrated in the medial, lateral, and anterior regions of the talar cartilage.

In this study, we developed a high-fidelity three-dimensional finite element foot model that explicitly incorporates all individual bundles of the DL. This detailed representation enables a quantitative analysis of the specific biomechanical contributions of each bundle. Furthermore, the integration of plantarflexion, dorsiflexion, and internal rotation alongside traditional tests provides a more comprehensive evaluation of the DL’s role in multidirectional ankle stability. Based on our findings that individual DL bundles exhibit distinct supporting functions across different degrees of freedom, treating the DL as a single structure or simply subdividing it into superficial and deep layers is not sufficient to fully characterize its biomechanical function. Clinically, most current reconstruction methods that reconstruct only the TNL bundle using a single bony tunnel [39,40] cannot restore the normal kinematics of the original DL [41,42]. Our findings may provide insights for DL reconstruction by quantitatively assessing the stability contributions of various bundles. This analysis clarified the key roles of the TCL, TNL, ATTL, and dPTTL in different planes of motion. It established a “priority list” to guide ligament selection when comprehensive DL reconstruction is not feasible. Based on preoperative assessment of the primary directions of instability and the patient’s functional demands in different planes of motion, specific bundles can be selectively reconstructed or reinforced to achieve an individualized surgical strategy. In this context, the motion assessment technology proposed by Belmonte et al., combined with inertial sensors, could provide standardized support for identifying primary instability patterns [43].

We acknowledge certain limitations of the present study. First, the finite element model was based on data from a single subject, which may limit its generalizability to a broader population. Individual anatomical variations (such as bundle size, fiber orientation, and material properties) in the DL may modulate the relative contribution of each bundle. For instance, bundles with a greater thickness or higher elastic modulus can potentially assume a more dominant role in load-sharing. Similarly, variations in fiber orientation dictate the moment components sustained by each bundle during ankle motion. Therefore, although our model reveals the fundamental biomechanical trends, the specific quantitative results obtained in this study may vary due to individual anatomical differences. However, this approach allows for precise control of individual variables and permits unlimited modification of specific factors without compromising the specimen’s integrity. Such modeling techniques are widely used to improve understanding of underlying biomechanical mechanisms. Second, this study did not examine more complex loading conditions, such as gait; however, this basic single-degree-of-freedom loading pattern may yield more precise results and support a fundamental understanding of the DL function. Future research could establish representative models categorized by distinct anatomical variations to investigate the influence of such diversity on these biomechanical findings. Furthermore, by coupling active muscle forces into the finite element model, it would be possible to evaluate how muscles and the DL synergistically maintain ankle stability under complex conditions such as gait.

## 5. Conclusions

The individual bundles of the DL were observed to contribute differently to ankle stability and articular contact pressure across various degrees of freedom. Within the specific anatomical configuration of the current model, the ATTL demonstrated a predominant role in maintaining anterior translational stability and in controlling articular contact pressure under anterior drawer loading. The TNL demonstrated a substantial contribution to internal–external rotational stability and plantarflexion stability, appearing crucial to maintaining articular contact pressure under these conditions. The TCL was found to be the primary structure for maintaining eversion stability and articular contact pressure during eversion. Furthermore, our results suggest that the dPTTL serves as a major structure for maintaining dorsiflexion stability and articular contact pressure.

## Figures and Tables

**Table 1:** Definition of material properties in the finite element model [24,27].

Components	Material Type		Young’s Modulus (MPa)			Poisson’s Ratio	
Bone	Linearly elastic		7300			0.3	
Cartilage	Linearly elastic		45			0.42	
Anterior talofibular ligament (ATFL)	Linearly elastic		255.5			0.4	
Calcaneofibular ligament (CFL)	Linearly elastic		512			0.4	
Posterior talofibular ligament (PTFL)	Linearly elastic		216.5			0.4	
Anterior tibiofibular ligament (ATiFL)	Linearly elastic		260			0.4	
Posterior tibiofibular ligament (PTiFL)	Linearly elastic		260			0.4	
Talonavicular ligament (TNL)	Linearly elastic		320.7			0.4	
Tibiospring ligament (TSL)	Linearly elastic		184.5			0.4	
Tibiocalcaneal ligament (TCL)	Linearly elastic		512			0.4	
Anterior tibiotalar ligament (ATTL)	Linearly elastic		184.5			0.4	
Superficial posterior tibiotalar ligament (sPTTL)	Linearly elastic		99.5			0.4	
Deep posterior tibiotalar ligament (dPTTL)	Linearly elastic		99.5			0.4	
Plantar fascia	Linearly elastic		350			0.35	
Other ligaments	Linearly elastic		260			0.35	
Bulk soft tissue	Hyperelastic: second-order polynomial strain energy potential expression						
	C_10_ 0.08556	C_01_ 0.05841	C_20_ 0.03900	C_11_ 0.02319	C_02_ 0.00851	D_1_ 3.65273	D_2_ 0.00000

## Data Availability

The data that support the findings of this study are available on request from the corresponding authors.

## Abbreviations

The following abbreviations are used in this manuscript:

DL	Deltoid ligament
ATFL	Anterior talofibular ligament
PTFL	Posterior talofibular ligament
CFL	Calcaneofibular ligament
ATiFL	Anterior tibiofibular ligament
PTiFL	Posterior tibiofibular ligament
TNL	Talonavicular ligament
TSL	Tibiospring ligament
TCL	Tibiocalcaneal ligament
ATTL	Anterior tibiotalar ligament
sPTTL	Superficial posterior tibiotalar ligament
dPTTL	Deep posterior tibiotalar ligament
